# The association between client type and condom use with steady and unsteady partners among persons seeking HIV testing and counseling services in Kenya

**DOI:** 10.4314/ahs.v17i4.5

**Published:** 2017-12

**Authors:** Elizabeth Broel, Larissa Brunner Huber, Jan Warren-Findlow, Elizabeth Racine

**Affiliations:** University of North Carolina at Charlotte, Department of Public Health Sciences

**Keywords:** Client type, condom use, HIV testing, Kenya

## Abstract

**Background:**

Approximately 70% of global HIV infections are located in sub-Saharan Africa, and the prevalence of HIV infection in Kenya remains high.

**Objectives:**

This study examined the association between client type (general population, commercial sex worker [CSW], or truck driver) and consistent condom use with steady and unsteady partners.

**Methods:**

Self-reported data included in the Kenyan Ministry of Health 2010–2011 National HIV Testing and Counseling Registry were used (n=11,567). Odds ratios (ORs) and 95% confidence intervals (CIs) were obtained using logistic regression.

**Results:**

After adjustment, CSWs and truck drivers had decreased odds of consistent condom use with steady partners compared to the general population (OR=0.52; 95% CI: 0.41–0.67 and OR=0.29; 95% CI: 0.13–0.63; respectively). CSWs had 1.95 times the odds of consistent condom use (95% CI: 1.58–2.42) and truck drivers had 0.64 times the odds of consistent condom use with unsteady partners (95% CI: 0.45–0.91) compared to the general population.

**Conclusion:**

Although CSWs consistently use condoms with their unsteady partners, truck drivers do not consistently use condoms with any partners. Future HIV prevention efforts should target CSWs and truck drivers to increase consistent condom use with all partners. Such efforts may decrease the prevalence of HIV in Kenya.

## Introduction

The prevalence of Human Immunodeficiency Virus (HIV) and Acquired Immunodeficiency Syndrome (AIDS) is disproportionately high in sub-Saharan Africa, with nearly 70% of the new global HIV infections located in this region^1^. While the overall estimated incidence rates of HIV/AIDS declined over 25% in 22 sub-Saharan African countries between 2001 and 2009, the estimated HIV/AIDS rates in Kenya remained stable^2^. In 2014, there were an estimated 1.4 million people living with HIV/AIDS in Kenya^3^.

Kenya has experienced a generalized epidemic of HIV/AIDS among the mainstream population and a more concentrated epidemic among most at-risk populations (MARPs)^4^. MARPs include commercial sex workers (CSWs), men who have sex with men, injectable drug users, and clients of commercial sex workers such as long distance truck drivers and military personnel^5^. According to the 2007 Kenya AIDS Indicator Survey, MARPs account for nearly one-third of all new HIV infections^6^.

Under the Kenyan National AIDS Strategic Plan 2009/10–2012/13, a multifaceted intervention strategy aimed at MARPs was developed since these individuals experience a highly negative social stigma and face barriers to healthcare access^4^–^6^. The goal of this intervention strategy was to increase access to HIV and reproductive health services for MARPs and ultimately decrease the incidence and prevalence of HIV among MARPs^6^.

Promoting condom use is an important component of MARPs HIV interventions. Consistent condom use can decrease the incidence of HIV up to 80%^7^, and studies have shown an increase in condom use following interventions targeted at condom use by as much as 70% among CSWs^8^. Numerous studies have been conducted in a variety of countries where HIV incidence is high examining social determinants in relation to risky behaviors, including condom use^9^–^27^. However, to date there have been no studies to compare condom use between multiple populations, specifically MARPs (CSWs and truck drivers) and the general population in Kenya, and no studies have compared condom use among steady and unsteady partners for both CSWs and truck drivers across multiple provinces in Kenya. Additionally, previous studies comparing condom use among steady and unsteady partners contained relatively small samples^11^,^12^,^17^. This study examined a large sample of participants seeking HIV Testing and Counseling (HTC) services using National HTC data from three provinces in Kenya to determine if CSWs, truck drivers, and the general population differed in their use of condoms with steady and unsteady partners.

## Methods

This cross-sectional study used data from the Kenyan Ministry of Health 2010–2011 National HTC Registry. The Kenyan Ministry of Health developed a national strategic plan (1999–2004) in response to the HIV epidemic in Kenya, and introduced comprehensive and standardized National HTC operations into the public healthcare system^28^. National HTC centers were developed to target those not yet infected with HIV, and to identify those infected with HIV and provide proper care^28^. Both static and mobile HTC models are available. Static sites include: integrated HTC centers located in the same structure as other physical health services; stand-alone HTC centers not associated with healthcare facilities; HTC centers connected to ongoing community or church programs; and HTC centers targeting special needs groups such as youth, MARPs, rape victims, and disabled persons. Mobile or outreach sites include workplace or home based counselling; vehicles with private counselling rooms; temporary tents; utilization of pre-existing facilities; and other mechanisms such as bicycles or camels^4^. Client HTC registration collects information about demographics, risky sexual behavior, HIV and other test results, distribution of condoms, and referral recommendations. Utilization of HTC services is strictly voluntary and informed consent is obtained prior to delivery of any HTC services^28^.

Data were collected from clients visiting HOPE Worldwide Kenya HTC centers between January 2010 and April 2011; ultimately, data from three provinces (Nairobi, Central, and Western provinces) were provided for analysis. This data represents both mobile and static sites. MARPs used mobile sites and the general population used both mobile and static sites. All HTC clients complete the standardized National HTC registration form at the registration desk^28^. The client then meets with a counselor to discuss the reason for visiting the HTC center and to evaluate HIV risk factors, including risky sexual behavior^28^. All data are entered into a database by trained data entry clerks^28^. All MARPs are issued a Unique Identifying Number and entered into a database dedicated to MARPs. General population data are entered into a separate database. The registration form and Unique Identifying Numbers are designed to ensure that individuals are included in only one database. However, it is possible that clerical errors may have resulted in a few individuals being included in both databases due to changes in MARP status during the study period.

A total of 11,567 individual records were included in the dataset (general population, n=10,547; CSWs, n=773; truck drivers, n=247). For the client type-consistent condom use with steady partners analysis, participants were excluded if they were <14 years of age or >80 years of age (n=79), had not been sexually active within the past 12 months (n=1,027), did not answer questions about condom use (n=21), or indicated that they did not have a steady partner (n=1,386). In addition, individuals missing information on education (n=40), marital status (n=27), knowing HIV status (n=24), and type of client session (n=9) were excluded. Thus, 8,954 participants remained for this analysis. For the client type-consistent condom use with unsteady partners analysis, individuals were excluded if any of the following applied: <14 years of age or >80 years of age (n=79), had not been sexually active within the past 12 months (n=924), did not answer questions about condom use (n=40), or indicated that they did not have an unsteady partner (n=6,014). Individuals missing information on education (n=42), marital status n=18), knowing HIV status (n=15), and type of client session (n= 5) were also excluded. A total of 4,448 participants were included in this analysis.

## Exposure assessment

Type of HTC client was the main exposure in this study. MARPs assessed in this study included CSWs and truck drivers. MARPs can be identified several ways: through self-report while completing the HTC registration form; by counselors while discussing occupation or risky behavior; or by trained peer educators. Regardless of how the MARP is identified, they are issued a Unique Identifying Number and their data are entered into a separate database dedicated to MARPs. HTC clients were classified into three categories for the purposes of this study: general population (non-MARP; referent), CSWs (MARP), and truck drivers (MARP).

## Outcome assessment

The main outcome of interest was risky sexual behaviors; specifically, condom use with steady and unsteady partners. Information regarding condom use was collected on the National HTC registration forms, and assessed during the consultation with a trained counselor, by querying about condom use in the last 12 months with steady and unsteady partners. Participants who answered that they did not have a steady/unsteady partner, had not had sex in the last 12 months, or had never had sex were excluded from the analysis as appropriate. Participants who indicated they always used condoms were considered to consistently use condoms while individuals who never or sometimes used condoms were considered to not use condoms consistently.

## Covariate assessment

Information on demographic and lifestyle characteristics collected on the National HTC registration forms were considered as potential confounding factors. Specifically, the following potential confounders were included: age, sex, education, marital status, knowing HIV status (i.e. in response to the question “Has client had an HIV test before?” the options were “No”; “Yes, Negative”; “Yes, Positive”; and “Yes, Do Not Know Result”), and whether the client came alone or as a couple^29^–^32^.

## Statistical analyses

Frequencies and percentages for all demographic and lifestyle characteristics were summarized for all participants according to exposure status. Logistic regression was used to calculate unadjusted odds ratios (ORs) and 95% confidence intervals (CIs) to assess the crude association between each type of HTC client and condom use with steady and unsteady partners. Additionally, other factors associated with condom use with steady and unsteady partners were identified. Multivariate logistic regression was used to calculate adjusted ORs and 95% CIs. Covariates were included in the model one at a time to determine if confounding was present. A covariate was considered a confounder if the magnitude of the OR changed by >10%^34^. All analyses were performed using SAS (version 9.2; SAS Institute Inc., Cary, North Carolina).

## Results

### Steady partners

Of the participants with steady partners, 90.8% were among the general population, 6.9% were CSWs, and 2.3% were truck drivers (Table 1). Approximately 78% of the general population and 63% percent of CSWs knew their HIV status, whereas only half (49.3%) of truck drivers knew their HIV status. Most participants did not use condoms consistently with steady partners; 84.7% of the general population, 86.5% of CSWs, and 96.6% of truck drivers were inconsistent condom users.

**Table 1:** Characteristics of Clients With Steady Partners utilizing HOPE Worldwide Kenya HTC Services, 2010–2011

Variables	General Population (N=8131) N (%)^a^	CSWs (N=620) N (%)	Truck Drivers (N=203) N (%)
**Age**			
14–20 yrs	378 (4.7)	16 (2.6)	1 (0.5)
20–29 yrs	4,958 (26.4)	384 (61.9)	65 (32.0)
30–39 yrs	647 (8.0)	43 (6.9)	45 (22.2)
≥40 yrs	2,148 (61.0)	177 (28.6)	92 (45.3)

**Sex**			
Female	3,034 (37.2)	620 (100.0)	4 (2.0)
Male	5,097 (62.7)	0 (0)	199 (98.0)

**Education Level**			
None or Some Primary	2,202 (27.1)	376 (60.7)	114 (56.2)
Some Secondary	3,742 (46.0)	212 (34.2)	82 (40.4)
Some Post-Secondary	2,187 (26.9)	32 (5.2)	7 (3.5)

**Marital Status**			
Never Married	1,264 (15.6)	240 (38.7)	30 (14.8)
Steady partner, living or not living together	2,554 (31.4)	108 (17.4)	18 (8.9)
Married	4,013 (49.4)	49 (7.9)	138 (68.0)
Separated/Divorced/Widowed	300 (3.7)	223 (36.0)	17 (3.2)

**Client Seen As**			
Individual	4,769 (58.7)	541 (87.3)	147 (72.4)
Couple	844 (10.4)	0 (0)	0 (0)
Group	2,518 (31.0)	79 (12.7)	56 (27.6)

**Know HIV Status**			
No	1,817 (22.4)	230 (37.1)	103 (50.7)
Yes	6,314 (77.7)	390 (62.9)	100 (49.3)

**Consistent Condom use in the** **last 12 months with Steady** **Partner**			
Yes	1,243 (15.3)	84 (13.6)	7 (3.5)
No	6,888 (84.7)	536 (86.5)	196 (96.6)

a Percentages may not equal 100 due to rounding

Compared to participants who were never married, individuals who were married, or were separated, divorced or widowed had statistically significant decreased odds of consistent condom use with steady partners (OR=0.08, 95% CI: 0.07–0.10, and OR=0.50, 95% CI: 0.39–0.65, respectively; Table 2). Participants who knew their HIV status had a 17% increased odds of consistent condom use with steady partners (OR=1.17, 95% CI: 1.02–1.35) than participants who did not know their HIV status. Both CSWs and truck drivers had decreased odds of consistent condom use with steady partners as compared to the general population, however, only the finding for truck drivers was statistically significant (OR=0.87, 95% CI: 0.69–1.10 and OR=0.20, 95% CI: 0.09–0.42, respectively). After adjusting for age and marital status, the client type-consistent condom use with steady partners association remained relatively unchanged for truck drivers and increased in magnitude for CSWs. Specifically, CSWs had 48% reduced odds (95% CI: 0.41–0.67; Table 3) and truck drivers had 71% reduced odds (95% CI: 0.13–0.63) of consistently using condoms with their steady partners as compared to the general population.

**Table 2:** Unadjusted Odds Ratios (OR) and 95% Confidence Intervals (CI) of the Association Between Select Characteristics and Consistent Condom Use With Steady Partners, 2010–2011 HOPE Worldwide Kenya HTC

	Unadjusted OR	95% CI
**HTC Client Type**		
General Population	1.00	Referent
CSWs	0.87	0.69, 1.10
Truck Drivers	0.20	0.09, 0.42

**Age**		
14–20 yrs	1.97	1.57, 2.46
20–29 yrs	1.00	Referent
30–39 yrs	0.22	0.15, 0.31
≥40 yrs	0.40	0.34, 0.47

**Sex**		
Female	1.00	Referent
Male	1.10	0.98, 1.24

**Education Level**		
None or Some Primary	1.00	Referent
Some Secondary	1.06	0.92, 1.22
Some Post-Secondary	1.43	1.23, 1.67

**Marital Status**		
Never Married	1.00	Referent
Steady partner, living together or not living together	0.98	0.85, 1.13
Married	0.08	0.07, 0.10
Separated/Divorced/Widowed	0.50	0.39, 0.65

**Client Seen As**		
Individual	1.00	Referent
Couple	1.73	1.45, 2.07
Group	0.96	0.84, 1.09

**Know HIV Status**		
No	1.00	Referent
Yes	1.17	1.02, 1.35

**Table 3:** Adjusted Odds Ratios (OR) and 95% Confidence Intervals (CI) of the Association Between HTC Client Type and Consistent Condom Use Among Steady Partners, 2010–2011 HOPE Worldwide Kenya HTC

	Adjusted OR^b^	95% CI
**HTC Client Type**		
General Population	1.00	Referent
CSWs	0.52	0.41, 0.67
Truck Drivers	0.29	0.13, 0.63

b Adjusted for age and marital status.

### Unsteady partners

Among participants with unsteady partners, 82.9% were among the general population, 13.6% were CSWs, and 3.5% were truck drivers (Table 4). Similar to participants with steady partners, nearly three-fourths (73.3%) of the general population and two-thirds (62.6%) of CSWs knew their HIV status, whereas only about half (49.4%) of the truck drivers knew their HIV status. Approximately 62% of the general population, 51.2% of CSWs, and 70.5% of truck drivers did not consistently use condoms with unsteady partners.

**Table 4:** Characteristics of Clients With Unsteady Partners utilizing HOPE Worldwide Kenya HTC Services, 2010–2011

Variables	General Population (N=3687) N (%)^a^	CSWs (N=605) N (%)	Truck Drivers (N=156) N (%)
**Age**			
14–20 yrs	206 (5.6)	13 (2.2)	1 (0.6)
20–29 yrs	2,395 (65.0)	373 (61.7)	58 (37.2)
30–39 yrs	215 (5.8)	44 (7.3)	31 (20.0)
≥40 yrs	871 (23.6)	175 (28.9)	66 (42.3)

**Sex**			
Female	859 (23.3)	605 (100)	3 (1.9)
Male	2,828 (76.7)	0 (0)	153 (98.1)

**Education Level**			
None or Some Primary	937 (25.4)	392 (64.8)	83 (53.2)
Some Secondary	1,694 (46.0)	187 (30.9)	70 (44.9)
Some Post-Secondary	1,056 (28.6)	26 (4.3)	3 (1.9)

**Marital Status**			
Never Married	1,158 (31.4)	279 (46.1)	39 (25)
Steady partner, living or not living together	1,010 (27.4)	70 (11.6)	11 (7.1)
Married	1,289 (35.0)	21 (3.5)	86 (55.1)
Separated/Divorced/Widowed	230 (6.2)	235 (38.8)	20 (12.8)

**Client Seen As**			
Individual	2,298 (62.3)	536 (88.6)	116 (74.4)
Couple	189 (5.1)	0 (0)	0 (0)
Group	1,200 (32.6)	69 (11.4)	40 (25.6)

**Know HIV Status**			
No	984 (27)	226 (37.4)	79 (50.6)
Yes	2,703 (73.3)	379 (62.6)	77 (49.4)

**Consistent Condom use in the** **last 12 months with Non-** **Steady Partner**			
Yes	1,405 (38.1)	295 (48.8)	46 (29.5)
No	2,282 (61.9)	310 (51.2)	110 (70.5)

a Percentages may not equal 100 due to rounding

CSWs had increased odds of consistent condom use with unsteady partners (OR=1.55, 95% CI: 1.30–1.84) and truck drivers had decreased odds of consistent condom use with unsteady partners (OR=0.68, 95% CI: 0.48–0.96; Table 5), as compared to the general population.

**Table 5:** Unadjusted Odds Ratios (OR) and 95% Confidence Intervals (CI) of the Association Between Select Characteristics and Consistent Condom Use With Unsteady Partners, 2010–2011 HOPE Worldwide Kenya HTC.

	Unadjusted OR	95% CI
**HTC Client Type**		
General Population	1.00	Referent
CSWs	1.55	1.30, 1.84
Truck Drivers	0.68	0.48, 0.96

**Age**		
14–20 yrs	0.74	0.55, 0.99
20–29 yrs	1.00	Referent
30–39 yrs	1.05	0.82, 1.34
≥40 yrs	1.05	0.91, 1.21

**Sex**		
Female	1.00	Referent
Male	1.00	0.88, 1.13

**Education Level**		
None or Some Primary	1.00	Referent
Some Secondary	1.04	0.90, 1.20
Some Post-Secondary	1.03	0.88, 1.21

**Marital Status**		
Never Married	1.00	Referent
Steady partner, living together or not living together	0.90	0.77, 1.06
Married	0.88	0.76, 1.03
Separated/Divorced/Widowed	1.09	0.89, 1.34

**Client Seen As**		
Individual	1.00	Referent
Couple	1.16	0.86, 1.56
Group	1.05	0.92, 1.20

**Know HIV Status**		
No	1.00	Referent
Yes	1.07	0.94, 1.22

After adjusting for sex, the client type-consistent condom use with unsteady partners association increased in magnitude for CSWs and remained largely unchanged for truck drivers. CSWs had 95% increased odds (95% CI: 1.58–2.42; Table 6) of consistent condom use with unsteady partners and truck drivers had 36% decreased odds (95% CI: 0.45–0.91) of consistent condom use with unsteady partners as compared to the general population.

**Table 6:** Adjusted Odds Ratios (OR) and 95% Confidence Intervals (CI) of the Association Between HTC Client Type and Consistent Condom Use Among Unsteady Partners, 2010–2011 HOPE Worldwide Kenya HTC

	Adjusted OR^c^	95% CI
**HTC Client Type**		
General Population	1.00	Referent
CSWs	1.95	1.58, 2.42
Truck Drivers	0.64	0.45, 0.91

c Adjusted for sex.

## Discussion

This study of consistent condom use among HTC clients in Kenya found that truck drivers were less likely to consistently use condoms with both steady and unsteady partners as compared to the general population. In comparison, CSWs were less likely to use condoms consistently with steady partners and more likely to use condoms consistently with unsteady partners as compared to the general population.

These findings are consistent with previous studies examining condom use among CSWs and truck drivers^10^,^11^,^29^,^34^,^35^. Voeten et al., examined sexual behaviors of CSWs in the Western province of Nyanza^11^. That study also found that condom use among CSWs was higher with unsteady partners than steady partners in both urban and rural regions of Kenya. Similarly, Gallo et al. investigated determinants of condom use among 140 Kenyan CSWs via a secondary data analysis using questionnaires from a study designed to investigate the safety and acceptability of diaphragms^10^. Researchers found that CSWs consistently used condoms 61% of the time with other (unsteady) partners and 28% of the time with regular (steady) partners.

Furthermore, Araoye et al. examined condom use among Nigerian truck drivers using a cross-sectional survey of 180 randomly selected drivers and found that 60% of Nigerian truck drivers were unwilling to use condoms even though 50% engaged in risky behaviors^34^. Sumnola examined sexual practices and condom use among truck drivers in Nigeria at eight major truck stops using a cross-sectional survey^29^. Sumnola found that, although 68% of truck drivers knew condoms help protect against HIV and STIs, only 32% actually used condoms. Mosoko et al. evaluated risk factors associated with HIV infection in a cross-sectional analysis among five specific population sub-groups in Cameroon, including CSWs and truck drivers, and found that 23% of CSWs and 37.9% of truck drivers did not regularly use condoms with unsteady partners^35^.

The results of this study should be interpreted in the context that all studies have strengths and limitations. Because the participants in this study all attended HTC centers on a voluntary basis, selection bias might have occurred because people who choose to utilize HTC services could differ from those people who choose not to utilize these services. Sex work, HIV^+^ status, and condom use are highly stigmatized in Kenya^36^ so people who choose to utilize HTC services may be more likely to use condoms than those who choose not to utilize HTC services. However, multiple studies have demonstrated condom use rates in sub-Saharan African countries similar to the rates in this study. Furthermore, according to the 2008–2009 Kenya Demographic Health Survey, 15% of the general population used condoms with steady partners and 48% of the general population used condoms with unsteady partners^37^. The similarities in the prevalence of condom use among the general population as assessed in the Kenya Demographic Health Survey and the general population in this study suggest that selection bias may be limited. The use of condoms was self-reported by all participants in this study, making the study more prone to recall bias. CSWs could potentially report a more socially desirable use of condoms to the HTC counselors as they know they should be using them. Having to recall condom use over a 12 month period could have also affected the outcome. Additionally, steady and unsteady sexual partners are self-reported. Kenya is a deeply religious country^38^ and those who participate in transactional or casual sex are often marginalized and stigmatized^36^. Due to the sensitive nature of the information being collected, information bias could have occurred and resulted in an underestimation of the true association.

Non differential misclassification of the exposure was also possible. National HTC centers put forth efforts to create safe, accessible, and confidential services for all clients and all clients are counseled by trained personnel as to the reason for the visit and their sexual behaviors^5^. While all HTC centers strive to maintain consistent protocols^28^, the experiences of clients may differ between different HTC centers. The classification of clients into the appropriate risk category (general population, CSW, or truck driver) is contingent upon the clients self-identifying their status, and MARPs may not always reveal their true occupation. Non differential misclassification of the outcome was also possible as the condom use outcomes rely on data that are self-reported by the participants. Having to recall condom use over the past 12 months could be difficult for some individuals. Lastly, this study was limited to the questions asked on the HTC registration forms. Thus, confounding due to known or unknown variables is possible.

This study also had several strengths. While studies have been conducted across multiple sub-Saharan African countries to examine condom use among MARPs, to date no study comparing condom use for both steady and unsteady partners has been conducted among MARPs in Kenya. Additionally, there have been no studies examining truck drivers in Kenya nor have there been any studies comparing CSWs and truck drivers to the general population in Kenya. Furthermore, there have been no studies in Kenya with a sample size larger than approximately 500 participants, encompassing more than one province, or directly comparing condom use between CSWs and truck drivers for both steady and unsteady partners. This study addressed all those gaps in the literature. Given the large sample size and the use of data from three of the eight major provinces in Kenya, the results of this study may be generalizable to the sexually active population seeking HTC services within Kenya.

## Conclusion

While additional research is needed to understand consistent condom use among MARPs, the information from this study can be useful for public health practitioners and HTC administrators in Kenya. By knowing which sub-populations of MARPs are consistently using condoms, practitioners will know which populations need to be targeted for continuing HIV prevention efforts. While the majority of CSWs are using condoms consistently with unsteady partners, intervention efforts need to focus on also promoting consistent condom use with steady partners. Additionally, public health practitioners should target interventions to truck drivers to promote consistent condom use with both steady and unsteady partners. Because knowing one's HIV status is a cornerstone of HIV prevention, the association between knowing HIV status and consistent condom use also has important public health implications for continuing HIV prevention efforts^39^. Intervention efforts combining promotion of consistent condom use and knowing one's HIV status may be a more effective method of HIV prevention.

Future research should focus on consistent condom use among all MARPs, including intravenous drug users and men who have sex with men, and barriers to condom use and HIV testing. This study dichotomized condom use; future condom use studies should consider a more indepth measurement of condom use to better understand the patterns of use with all sexual partners. By understanding factors associated with consistent condom use among sub-populations within Kenya, public health practitioners and HTC administrators can continue to develop a multi-faceted approach to HIV education and prevention in hopes of decreasing the overall prevalence of HIV in Kenya.
